# Go Where the Students Are: A Comparison of the Use of Social Networking Sites Between Medical Students and Medical Educators

**DOI:** 10.2196/mededu.4908

**Published:** 2015-09-08

**Authors:** Safaa El Bialy, Alireza Jalali

**Affiliations:** ^1^ Division of Clinical and Functional Anatomy Department of Innovation in Medical Education University of Ottawa Ottawa, ON Canada

**Keywords:** social media, e-learning, innovations in medical education

## Abstract

**Background:**

Medical education has grown beyond the boundaries of the classroom, and social media is seen as the bridge between informal and formal learning as it keeps students highly engaged with educational content outside the classroom.

**Objective:**

The purpose of this study is to explore the perceptions of medical educators and medical students regarding the use of social media for educational purposes.

**Methods:**

Both groups (medical educators and students) were invited to take a survey. The surveys consisted of 29 questions, including Likert-style, multiple choice, yes/no, ranking, and short answer questions. The survey forms and statistics were built using Google Drive analytics with the free Spanning Stats module. To compare between professors and students, results were exported to a Microsoft Office Excel spreadsheet (Microsoft Corp, Redmond, WA). The study protocol was approved by The Ottawa Health Science Network Research Ethics Board (OHSN-REB:20140680-01H).

**Results:**

The overall response rate to the survey was 40.9% (63/154) for students, and 36% (72/200) for medical educators. The majority of educators (79%, 57/72) and students (100.0%, 63/63) had presence on social networking sites (SNSs). Only (33% 19/57) of educators used SNSs with their students, the most used sites were Facebook (52%, 10/19) and Twitter (47%, 9/19), followed by LinkedIn (21%, 4/19), Google+ (16%, 3/19),YouTube (11%, 2/19), and blogs (11%, 2/19). Facebook (100%, 63/63), YouTube (43%, 27/63), Twitter (31%, 20/63), and Instagram (30%, 19/63) were the sites most commonly used by students. The educators used SNSs mainly to post opinions (86%, 49/57), share videos (81%, 46/57), chat (71%, 41/57), engage in medical education (68%, 40/57), take surveys (24%, 14/57), and play games (5%, 3/57). On the other hand, students used SNSs mainly to chat with friends (94%, 59/63), for medical education purposes (67%, 42/63), to share videos (62%, 39/63), to post opinions (49%, 31/63), to take surveys (11%, 7/63), and to play games (6%, 4/63). Most educators (67%, 38/57) do not use social media in their education Although most of the educators (89%, 17/19) and students (73%, 46/63) found the use of social media time-effective, that it offered an inviting atmosphere (89%, 17/19 and 70%, 44/63), and that it enhanced the learning experience (95%, 18/19 and 70%, 44/63), both groups stated that they had colleagues who refused to use social media. The detractors’ concerns included privacy issues (47%, 18/38), time-wasting (34%, 13/38), distraction (21%, 8/38), and that these media might not be suitable for education (11%, 4/38). When it came to using SNSs with the students, the educators most often used SNSs to post articles (42%, 8/19), explanatory comments (31%, 6/19), and videos (27%, 5/19).While students preferred the following posts : Quizzes (87% 55/63), revision files (82% 52/63) and explanatory comments (29% 21/63).

**Conclusions:**

Although social media continue to grow, some educators find that they do not offer suitable modes of learning. However, it is important to acknowledge that there are persistent differences in technology adoption and use along gender, racial, and socioeconomic lines; this is often referred to as the “digital divide”. The current study shows that students prefer certain posts like quizzes and revision files, while educators are focused on posting videos, articles, and explanatory comments. Medical educators are encouraged to focus on the students in a way to minimize the gap between learners and educators. It will remain our responsibility as educators to focuson the student, use SNSs at their fullest, and integrate them into traditional Web-based management systems and into existingcurricula to best benefit the students.

## Introduction

Information and communication technologies (ICTs) have evolved tremendously over the last few decades, bringing with them a new perspective on methods of teaching and learning. Currently, social networks are used by millions of users, most commonly adolescents, for a variety of purposes, such as chatting, socializing, posting, and most interestingly, learning [1,2]. Medical learning has grown beyond the boundaries of the classroom and now social media is seen as the bridge between informal and formal learning as it keeps the students highly engaged with educational content when not in the classroom [3].

In recent years, new Web-based social media have been portrayed as placing the learner at the centre of knowledge networks by providing expertise that can potentially lead to new forms of learning despite the fact that these media make no educational promises. Educators have advocated integrating Facebook, Ning, and other sites into kindergarten to grade 12 (K-12) academic life [4]. Advocates have also promoted social media use as part of a connectivist learning theory. This recent move elevates social and communicative connections to the level of an epistemic category central to learning processes. In other words, social media learning empowers students [5,6]. This type of learning is characterized not only by greater autonomy for the learner but also by changing roles for the teacher; indeed, a collapse of the distinction between teacher and student altogether is indicative of connectivist learning [6].

Many teachers have adopted an everyday practice of incorporating digital technologies in the classroom and extending learning beyond the traditional boundaries of the institution. The rapid advance of technology is driving educators to implement tools they may have only recently learned. Most college age students, otherwise known as digital natives, Generation Y (Gen Y), Net Generation (Net Gen), and Millennials [7] are far ahead of teachers in technology usage and they are demanding that technology be used within the classroom. This younger generation of students has spent their entire lives surrounded by and using computers, video games, digital music players, video cameras, mobile phones, and all the other toys and tools of the digital age [8].

Incorporating digital technology into everyday study has changed not just the teacher’s role but also the way students learn [9]. Now students can assume more responsibility for their own learning, access a vast pool of knowledge, and learn anywhere at their own pace. As well, they can share knowledge and experiences, communicate with their peers and teachers, and enhance their learning experiences.

Experts in any field are more accessible than ever before, thanks to social media. They share their ideas and experiences in videos, articles, and so on. Thus, even before first encountering students, teachers can provide an insight into the professional world and prepare students for their studies. Students get the chance to "meet" their professors and their classmates even before arriving on campus.

One of the major advantages of social media tools is the creation of community. Social media fosters communication, engagement, and collaboration [10-12]. A community can be created locally for a particular class, university, or it can extend beyond a single campus using a virtual world, such as Second Life [12]. This way of communication is well-suited for the lives of college-age students, Millennials who were born in the technology age and who are very technology savvy, despite the debate that using technology does not necessarily mean that the user understands technology [13].

However, with the growing amount of time that youth are spending on social networking sites (SNSs), do educators consider these sites to be of educational value? The purpose of this study is to compare the use of social media as an educational tool by medical students versus medical educators.

## Methods

### Overview

The infrastructure of the Faculty of Medicine at the University of Ottawa is designed to allow free WiFi Internet access for all employees and students anywhere on the campus. At the University of Ottawa, online support for courses includes teaching materials that are available through a learning management system. The study protocol was approved by The Ottawa Health Science Network Research Ethics Board (OHSN-REB: 20140680-01H).

### Recruitment

Professors of different medical specialties (N=200) and second year medical students (N=154) were surveyed regarding their use of SNSs as an extracurricular way of enhancing the learning experience. Survey questions were based on the current literature [14-17] and optimized with input from faculty and medical student focus groups.

### Study Structure and Statistical Analysis

The surveys consisted of 29 questions including Likert-style, multiple choice, yes/no, ranking, and short answer questions. The survey forms and statistics were built using Google Drive analytics with the free Spanning Stats module.

The survey questions were divided into the following sections, each of which approached a concern about professors’ and students’ use of social media (1) social media presence, (2) purpose of social media use, (3) frequency of social media access, (4) the devices used for social media access, and (5) perceptions of social media use in education.

The frequency of social media use was the number of times the user accessed SNSs in a given hour, day, week, or month. With respect to the medium of social media access, laptop, desktop, mobile phone, and tablet were the devices listed. Medical educators and students were asked about their reasons for using social media in general and for educational purposes in particular. Respondents’ opinion about using social media in education were tested by asking them to rate on a Likert scale, ranging from strongly disagree to strongly agree (Textbox 1).

In addition, participants were asked about their concerns with using social media in education. To compare professors and students, results were exported to a Microsoft Office Excel spreadsheet (Microsoft Corp, Redmond, WA).

**Statements rated on a Likert scale.**

Statement

1. Social media can be used as a suitable learning environment.
2. Social media sites are more accessible than other ways of communication.
3. I am aware of privacy settings that limit access to personal information.
4. Social media provide an inviting atmosphere that encourages me to participate.

## Results

The overall response rate to the survey was 40.9% (63/154) for students and 36.0% (72/200) for medical educators.

### Medical Educators

Of the medical educators who participated, most were clinicians (52%, 37/72), full-time professors (26%, 19/72), part-time professors (13%, 9/72), and others (10%, 7/72) (eg, teaching assistants and sessional lecturers), and the majority dedicated more than 100 hours per year to their teaching (55%, 40/72). Some participants (21%, 15/72) did not have a presence on any SNSs; among those who did, the most frequently used sites were Facebook (61%, 35/57), LinkedIn (47%, 27/57), Twitter (46%, 26/57), Google+ (30%, 17/57), and YouTube (22%, 12/57), followed by other sites including Pinterest, Instagram, and Tumblr (Table 1).

**Table 1:** Students and medical educators social networking sites (SNSs) use by type of media.

SNSs	Users, n (%)	
	Medical students (n=63)	Medical educators (n=57)
Facebook	63 (100%)	35 (61%)
YouTube	27 (43%)	12 (22%)
Twitter	20 (31%)	26 (46%)
LinkedIn	13 (21%)	27 (47%)
Instagram	19 (30%)	5 (8%)
Pinterest	11 (18%)	10 (18%)
Google+	8 (13%)	17 (30%)
Reddit	5 (8%)	1 (2%)
Tumblr	4 (6%)	1 (2%)

The educators used SNSs mainly to post opinions (86%, 49/57), share videos (81%, 46/57), chat (71%, 41/57), engage in medical education (68%, 40/57), take surveys (24%, 14/57), and play games (5%, 3/57) (Table 2). Most educators (67%, 38/57) did not use social media in their education. They expressed concerns such as privacy issues (47%, 18/38), time-wasting (34%, 13/38), distraction (21%, 8/38), and that these media might not be suitable for education (11%, 4/38). Some educators even mentioned that they were unaware of social media use in education (29%, 11/38).

**Table 2:** Students' and medical educators' use of social networking sites (SNSs).

Uses of SNS	Users, n (%)	
	Medical students (n=63)	Medical educators (n=57)
Medical education	42 (67%)	13 (23%)
Play games	4 (6%)	3 (5%)
Chat	59 (94%)	45 (71%)
Share videos	39 (62%)	40 (70%)
Post opinions	31 (49%)	54 (95%)
Take a survey	7 (11%)	15 (26 %)

Of the educators who used SNSs with their students, the most used sites were Facebook (52%, 10/19) and Twitter (47%, 9/19), followed by LinkedIn (21%, 4/19), Google+ (16%, 3/19), YouTube (11%, 2/19), and blogs (11%, 2/19). About 32% (6/ 19) of educators logged into their accounts daily, 21% (4/19) logged in a few times per week, 5% (1/19) logged in weekly, and another 5% (1/19) logged in a few times per month. Finally, 21% (4/19) logged in only once per month and 16% (3/19) rarely logged into their accounts (Table 3).

When it came to using SNSs with the students, the educators most often used SNSs to post articles (42%, 8/19), explanatory comments (31%, 6/19), and videos (27%, 5/19). Others used SNSs to post lecture comments (31%, 6/19), book recommendations (31%, 6/19), revision files (21%, 4/19), quizzes (8%, 3/19), and course related humor (16%, 3/19). Most participants (95%, 18/19) would recommend SNSs to their colleagues, and 80% (15/19) found social media to be more accessible than other ways of communication. Strong majorities also considered social media to be time effective (89%, 17/19), inviting (89%, 17/19), and an improvement in the learning experience (95%, 18/19). In addition, 79% (15/19) of participants stated that they had colleagues who refuse to use social media for educational purposes.

**Table 3:** Students and medical educators' frequency of logging into social networking sites (SNSs).

Frequency of logging into SNSs	Users, n (%)	
	Medical students (n=63)	Medical educators (n=19)
Lost count	21 (33%)	
Few times a day	32 (51%)	
Every hour	5 (8%)	
Few times per hour	5 (8%)	
Daily		6 (32%)
Few times per week		4 (21%)
Once per month		4 (21%)
Few times per month		1 (5%)
Rarely		3 (16%)

### Medical Students

All of the students (100%, 63/63) had presence on SNSs. The most common used sites were Facebook (100%, 63/63), YouTube (43%, 27/63), Twitter (31%, 20/63), and Instagram (30%, 19/63). Other social networking sites followed, as shown in Table 1.

The students used SNSs mainly to chat with friends (94%, 59/63), for medical education purposes (67%, 42/63), to share videos (62%, 39/63), to post opinions (49%, 31/63), to take surveys (11%, 7/63), and to play games (6%, 4/63) (Table 2).

The devices that students used to access their accounts were laptops (94%, 59/63), followed by mobile phones (70%, 44/63), tablets (33%, 21/63), and desktops (11%, 7/63). With respect to the frequency of social media access, 51% (32/63) of students logged into their accounts a few times per day and 33% (21/63) stated that they received push notifications, as the account is always running on their mobile devices. Another 8% (5/63) logged into their accounts daily and 8% (5/63) a few times a day (Table 3).

The types of educational posts that students preferred are shown in Table 4. Most students (77%, 50/63) would recommend SNSs to their peers and 62% (39/63) stated that they have friends who refuse to use these sites mainly because of distractions; privacy issues were not the main concern as 88% (55/63) of the students stated that they knew how to adjust privacy settings. Most students (73%, 46/63) found social media to be a time-effective way of communication and 70% (44/63) found it an inviting atmosphere that improved their learning experience. However, 68% (43/63) of students stated that no other professor used social media as a way of communication with their students.

**Table 4:** Students' preferred types of educational posts (n=63).

Type of post	n (%)
Quizzes	55 (87%)
Revision files	52 (82%)
Explanatory comments	21 (29%)
Post-lecture questions	14 (22%)
Course-related humor	14 (22%)
Book/article recommendations	14 (22%)
Videos	9 (14%)

## Discussion

### Principal Findings

According to Coates:

Blended learning, integrating a variety of media to deliver teaching material to students is used by universities all over the world. It is associated with the use of Web tools such as email, podcasts, blogs, discussion boards, and a university learning management system. [18]

These systems provide a Web presence for course instructors to manage the course material [18]. They also have the advantages of being student-centered, focused on the course material, no distractors, and no privacy issues. “No matter how those systems are developed to manage processes such as exams, assignments, course descriptions, and basic course material, they are not well-suited to some other activities such as problem-based learning” [19]. In addition, they lack the element of social connectivity which today’s students are familiar with [20].

The emergence of SNSs has raised questions about whether we should confine ourselves to course-integrated learning management systems or whether we should go where the students are. Educators do not want to integrate these tools into their curriculum just for the sake of technology [21]. However, today’s college-age students are the first generation to grow up with the Internet; they do not remember a time when it did not exist. They are technologically savvy and dependent upon the Internet. Therefore, educators must reach out and engage these students with social media and even join their communities or create similar ones [22,23].

The current study shows that there is a controversy about whether to use SNSs in general and in education. While 100% (63/63) of students had a presence on SNSs, only 79% (57/72) of medical educators were members of those sites and only 33% (19/57) used them with their students. These results are in accordance with the results of a survey conducted by the Babson Survey Research Group in collaboration with New Marketing Labs and the education-consulting group Pearson Learning Solutions. The survey was drawn from almost 1000 college and university faculty nationwide and found that more than 80% of professors used social media in some capacity. The survey noted that 30% of educators used social networks to communicate with students and more than 52% used online videos, podcasts, blogs, and Wikis during class time. It was also found that older faculty used social media at almost the same level as their younger peers [24] which contradicts our result that 67% (38/57) of medical educators who did not use SNSs in their teaching were in the older age group (age >50). Rank also subsumes age differences that exist among faculty; older people normally occupy the rank of associate or full professors. O’Shea [25] argues that the distinctions for adopting technologies are blurring the traditional dichotomies which now are best characterized as 5 groups of innovators, early adopters, early majority, late majority, and laggards. Indeed, one study found that age was a poor predictor of social media usage within a research context [26].

The use of social media is, however, increasing rapidly in the classroom [27]. Our study shows that Facebook, LinkedIn, and Twitter are the most popular sites for medical educators while for students, Facebook, YouTube and Twitter were the top 3 sites (Table 1). Educators used those sites mainly to post opinions, share videos, chat, and for medical education purposes, and students mainly used them to chat, for medical education purposes, to share videos, post an opinion, take a survey, or to play games (Table 2). For the 33% (19/57) of medical educators who used social media with their students, Facebook, LinkedIn, and Twitter were again the top 3 sites and they were mainly used to post articles, videos, and explanatory comments. Students, however, preferred the quizzes and the revision files.

There are several reasons that educators at the University of Ottawa were in favor of using SNSs with their students including (1) the ease of accessibility and connectivity with students, (2) the ease of disseminating information and accessing different audiences, (3) time flexibility and virtuality, (4) interactivity, (5) students’ reliable use of these sites, and (6) the ability to use the same material repeatedly.

The reasons students favor SNSs included (1) interactions with colleagues (asking/answering questions), (2) sharing resources, (3) accessibility, (4) post-lecture questions, (5) the ability to review quizzes before exams, and (6) the ease of tracking information.

Although most of the participants (medical educators and students) would recommend social media to their colleagues, the majority had colleagues who refuse to use SNSs for educational purposes. These colleagues were mainly concerned about privacy issues, distractions, and wasting time. Interestingly, some of the educators stated that they did not even know about social media use for educational purposes. Cheston et al [28] stated that social media tools can be used safely in medical education settings and that their use may have a positive impact on learner outcomes.

The results of the present study are in agreement with the results of Chen et al [29] who found that the use of social media in teaching is relatively scarce. Despite the limited use of social media in the academic world, research has supported the connectivism theory and found benefits of using social media if the technology is adapted for teaching [30,31]. Most faculties agree that the interactive nature of social media technologies create better learning environments and increase the communication with peers and students. However, concerns remain about distraction and privacy issues.

Our results contradict the results of Moran et al [32], who found that 90% of faculties used social media in courses they taught. Nearly two-thirds of all faculties in the Moran study used social media during a class session and 30% had posted content for students to view or read outside of class. Over 40% of faculty required students to read or view social media as part of a course assignment and 20% had assigned students to comment on or post to social media sites.

These findings demonstrate that SNSs are growing and are here to stay. However, it is important to acknowledge that there are persistent differences in social media use along gender, racial, and socioeconomic lines; the so-called digital divide [33], and those parameters need to be further explored.

### Conclusions

Today's students grew up with the Internet and they don’t remember a time where it did not exist. They are communicating, learning, and adapting to the world via this relatively new way of communication. Although social media continue to grow, some educators find that they do not offer a suitable mode of learning. However, the current study shows that students prefer certain posts like quizzes and revision files, while educators are focused on posting videos, articles, and explanatory comments. Medical educators are encouraged to triage the students at the beginning of the semester and find out what their preferences are and focus on them in a way to minimize the gap between learners and educators.

Despite all the criticism, social media have the potential to build interactivity, engagement, and collaboration. It will remain our responsibility as educators to focus on the student, use SNSs at their fullest, and integrate them into traditional Web-based management systems and into existing curricula to best benefit the students.

## Abbreviations

ICT: information and communication technology
SNS: social networking site
